# Community-Based Research among Marginalized HIV Populations: Issues of Support, Resources, and Empowerment

**DOI:** 10.1155/2012/601027

**Published:** 2012-09-10

**Authors:** Mario Brondani, Nardin R. Moniri, R. Paul Kerston

**Affiliations:** ^1^Faculty of Dentistry-Oral Health Science, University of British Columbia, 122/2199 Wesbrook Mall, Vancouver, BC, Canada V6T 1Z3; ^2^Faculty of Integrated Sciences, University of British Columbia, Vancouver, BC, Canada V6T 1Z3; ^3^Treatment Outreach and Community Representation & Engagement, Positive Living Society of British Columbia, Vancouver, BC, Canada V6B 5S8

## Abstract

A research question was posed to us by a local HIV-resource organization interested in exploring the educational and service needs of those unreached. In order to properly address this inquiry, we developed a community-based participatory research by training peer-led volunteers to facilitate focus-group discussions within Aboriginal and refugees participants following an interview guide. We gathered Aboriginal people and refugees separated into three focus groups each, enrolling a total of 41 self-identified HIV-positive, 38 males. The discussions were tape recorded upon consent and lasted between 59 and 118 minutes. We analyzed the thematic information collected interactively through constant comparison. The qualitative data leading to categories, codes, and themes formed the basis for the spatial representation of a conceptual mapping. Both groups shared similar struggles in living with HIV and in properly accessing local nonmedical HIV resources and discussed their concerns towards the need for empowerment and support to take control of their health.

## 1. HIV Resource Services and Marginalized Communities

The available literature has been quite prolific on issues around the medical side of HIV, particularly the uptake of HIV antiretroviral therapy adherence, cost, availability, and side effects [1–4]. Very few have discussed access and use of nonmedical HIV resources services [5]. Vancouver, Canada, has experienced a decrease in new HIV diagnoses [6], while new HIV cases amongst Aboriginal peoples and other populations continue to rise [7, 8]. New immigrants and refugees from HIV-endemic countries add to this pool when arriving to Canada [9, 10]. 

The strategic plan of the BC Center for Excellence in HIV/AIDS (BC-CfE) and reports from the BC Center for Disease Control have highlighted that different HIV-marginalized population groups in Vancouver remain at risk for further health struggles and isolation unmitigated by stigma and fear [5, 9]. Our understanding of marginalised communities encompass those who are disadvantaged in terms of the structures and conditions that shape their lives, health outcomes, and social positions in society [11]. In order to utilize the full advantage of HIV/AIDS information including printed media, support groups, and drop-ins, these marginalised groups must be aware of the existence of these resource services. However, there seems to be a gap between the resources that are being offered and those accessing them [2, 12]. Based on the current trend of HIV infection identified in the BCCDC and BC-CfE reports, different HIV population groups remain marginalized, including the following.

- *Aboriginal people* who make up approximately 5% of the population in BC but are disproportionately represented in BC's HIV epidemic. Aboriginal males comprised 14.6% of all new positive-HIV cases among males in 2009, while females comprised 23.9% of all new positive HIV cases among females for the same year.
- *HIV seropositive refugees* who arrive annually in Canada. In 2009, 46% of HIV seropositive immigrants who arrived in BC came from countries where HIV is considered to be endemic.

## 2. Community-Based Participatory Research

As a qualitative approach to research, community-based participatory research (CBPR) has become mainstream in health disciplines including nursing and social work [13], particularly on issues related to HIV [14–18]. CBPR implies a collaborative and equitable partnership among community members, researchers, and organizational representatives in all aspects of the research process [19]. Such participatory research is viewed as a relevant framework to promote empowerment and engagement towards meaningful action as it fosters openness and trust among different partners [20]. Maintaining trust and building strong bonds among the members are crucially valuable in maintaining the effectiveness of CBPR. In addition, each member brings strengths, values, and shared responsibilities towards unraveling a given social phenomena [21]. According to Minkler and Wallerstein [21], CBPR can encompass nine integrated and overlapping principles, from recognizing the community as the unit of identity for promoting capacity building and identifying a problem of local relevance. Community involvement has been key in working within the context of HIV/AIDS research to the extent that joined efforts have culminated the development of the Greater and Meaningful Involvement of People living with HIV (GIPA/MIPA) principles. Such principles bring together people living with HIV/AIDS in the decision-making processes that affect their lives and those around them, from family and friends to their communities. Those living with HIV help community organizations to identify and advocate for the needs of their peers along with ideas on how to implement strategies for further change while supporting and assisting the service users [22, 23]. In the case of our experience, the involvement of people living with HIV/AIDS prompted us to develop this study as one of the local HIV resource organization, as our unit of identity, told us that *“given the discrepancy between the demographics of HIV cases in Vancouver and the demographics of our current members, what are the educational and service needs of those we are not reaching?”* With this research question arising from the community itself, we engaged collaboratively in giving voice to Aboriginal individuals and refugees who are HIV seropositive in expressing their needs and their expectations of HIV-resource services in Vancouver, Canada. Although a through description of the initial set of findings from that study can be found at Brondani et al. [5], we aim at drawing from some of those findings on issues of empowerment, support, and resources allocation and utilization within supplemental focus groups discussions.

## 3. Methods

The previous and current research and volunteer partnerships established between the first author and members of local HIV-resource organizations were central to this community-based study and have created long-lasting collaborative initiatives and rapport. Such collaboration culminated with one of the community members coauthoring this paper (third author). Initially, we performed an environmental scan to identify key informants, other organizations, and health clinics that were likely to care for members of these specific groups in terms of their medical necessities and HIV medication. These organizations approved an advertisement to recruit potential participants for local audio-recorded focus group discussions [24]. We sought for HIV-positive self-identified individuals who were males and females 19 years and older of any sexual orientation. There was no medical confirmation of their HIV diagnosis, but self-report. Participants signed the informed consent which was approved by the community organizations involved and by the UBC Ethical Review Board # H11-00197. 

## 4. Focus Groups

We chose group discussion as they help to explore values and beliefs when little is known about the nature and complexity of the personal and social factors that affect perception of health needs. A focus group (FG) allows participants to explore feelings, experiences, opinions, and new ideas [25] while gathering information from a variety of participants at the same time as discussed by Kreuger [26]. Participants can question one another, explain and elaborate specific points, seek clarification, pose comments, and prompt the group to further refine the information generated [27, 28]. In stigmatized and marginalized groups such as those we were interested in listening to, group discussions can foster socialization and membership and generate transformative change [17, 29]. The implementation of FG in a community-based enterprise within marginalised communities requires closer attention to issues of power relationship and stigma as participants may express discomfort and anxiety [30, 31]. As a result, peer-led facilitators are suggested to create rapport and foster sense of belonging [32, 33], especially around HIV-related issues [34, 35]. We trained four peer volunteers, two per each of our target populations, to build on existing community resources as described at Brondani et al. [5]. Although the number of group discussions needed to explore a given topic is usually determined by the scope of the study, we proposed three FG per each population for a total of six at this stage.

## 5. Sample

Forty-one volunteer participants came forward to take part in group discussions during Spring and Summer of 2011. They anonymously filled out a form about their ethnocultural and educational backgrounds, marital status, and self-perceptions of general and oral health. Nineteen participants are self-identified as Aboriginal, all males between the ages of 25 and 51; seventeen were currently on HIV medication and thirteen self-rated their general heath as “good”. The other twenty-two participants were refugees from Mexico, Colombia, and Equador between the ages of 24 and 71 and 18 were males; fifteen were currently on HIV medication and seven self-rated their general health as “fair”. The six group discussions happened once, were tape-recorded upon consent, and lasted between 59 and 118 minutes. The three focus groups that involve refugees happened in Spanish. Participants from all groups were already utilizing some local medical-related services [36] but resources services to a much lesser extent. We believe that participants within these two groups represented a broad range of social-related characteristics, backgrounds, experiences, and beliefs [29]. However, we did not have an equal representation of females participants, and for that, results have to be examined with caution [37].

## 6. Data Analysis

**Figure 1:**
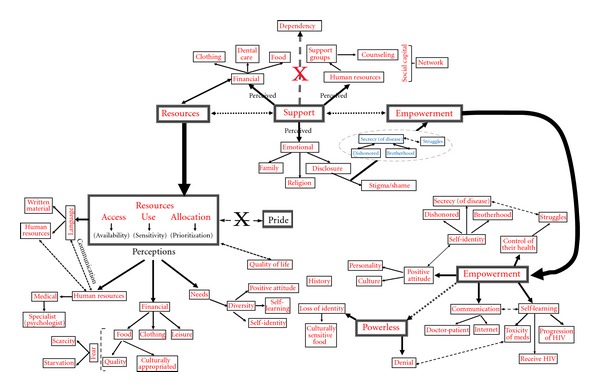
Relationship of themes and codes of the categories resources, support, and empowerment using mapping technique (adopted from [37]).

N-Vivo 8 software program (QSR) was used to help analyzing the group discussions thematically and inductively by the first author (MB). The coauthors gave comments and feedback on the interpretation of the data, compilation of the thematic clusters, and presentation of the findings in the final report and this paper. The codes, themes, and categories that emerged from the interactive constant comparison analysis were linked via maps using Power Point (Microsoft Office 2007) [37] in an attempt to represent their overlapping relationships as seen in Figure 1. Figure 1 is an amalgamation of the three main categories of empowerment, support, and resources that have been thoroughly discussed by Brondani et al. [5]. The thematic analysis was done according to the following process: essential codes (e.g., words signifying an attribute or denomination) are identified inductively from excerpts from the discussions (transcribed and translated) to assure the completeness, accuracy, and relevance of the categories and themes being explored. NVivo 8 software program for data management did not analyze the gathered information per se since the researcher assigned codes inductively. Although one single group discussion could be assigned various codes and various codes could be linked to different discussions, it was unlikely that a single group would cover all categories and themes. A 62-page final report was given to each of the organizations participating in this study (e.g., where the discussion took place) to enhance collaboration, accountability, and knowledge transfer. 

## 7. The Findings

The thematic breakdown of the codes, themes, and categories in Figure 1 is presented at the group rather than at individual level due to the interactive nature of the group discussions [25, 26, 38]. However, quotes from individual participants from different groups are used to illustrate specific issues and might represent agreement, as group members nodded, or a stand-alone comment that pertained to an individual and their unique experience.

Within the group discussions, three main categories emerged: empowerment, support, and resources. Empowerment referred to the willingness participants had in being included in the decision-making processes that affect their lives, while refuting the medical model approach to HIV/AIDS as an acquired disease in isolation from the person. Such approach has indeed been challenged by the GIPA and MIPA principles motivated by the need for this greater and more meaningful involvement of people living with HIV/AIDS. Fulfillment of this need was central to the development of our community-based participatory study. For empowerment, group members mentioned the idea of having services that were significant to their ethnocultural background within the idea of self-identity and brotherhood. For most of the Aboriginal participants, the issue was to *“offer more services for aboriginal people, having medical doctors who are First Nations themselves, as brother and sisters, more culturally relevant… we have to go to another place to get *[medical services] *and we do not have our own place.”* Such call echoes the statements put forward by the Canadian National Indian Brotherhood which emphasized the need for local control of integrated education and support for, and by, First Nations more than 40 years ago [39]. Although the Latino groups raised similar concerns, they also focused on language as a barrier (overlapping with Resources) as *“we do have good services in Vancouver, but it is all in English! Very few prints in Spanish about HIV and information in general. I know English is the language, but some explanations at this point have to be in a way I can understand clearly. Dr XX is Spanish, but because he is one of the very few, he is fully booked for months.” *In fact, Jimenez [40] highlighted the lack of language-specific information on how to deliver services to immigrant and refugees from HIV-endemic regions. Although services could be provided in respect to cultural norms [41], culturally related services were not a priority or a need, to all participants. One Aboriginal group member mentioned that *“services are to everybody… to anyone… the community has to be one, doesn't matter where you come… if you want to get better, you have to be positive about it, that is all what matters.”*

This view of positive attitude plays also an important role in dealing with the daily struggles with HIV as a hidden, secretive condition and counteracts the fear of being dishonoured by family and friends once the HIV status is known [42]. In fact, the positive attitude that people have in life in general and with their HIV status in particular as a personality trait plays an important role within people's lives. In the three groups of Aboriginal members, participants nodded in agreement when hearing their peers mention, for example, that *“each of us have [Sic] our own way to see things… my whole understanding is to continue to apply positive thinking.”* And that *“we learned throughout life how to deal with difficult situations and be proactive about them.”* Similarly, personality was a trait of one being positive or negative about life and heavily influenced by historical and current cultural norms [43, 44].

Electronic and verbal communication in general, and between doctors and the patients in particular, was perceived as being empowering in the a sense that individuals are able to get valuable information and proactively self-learn about their health and the progression of their disease [45–47]. Different participants expressed, however, struggling to come to terms with the fact that they have HIV, which may put them in a position of not being able to control their health. Such struggle might help explain why they disclose their HIV status to some people but keep it secret from others. The struggles of being HIV seropositive might be minimized when the individual knows that s/he has reached an undetectable viral load and a perceived good CD4 count, even though the impact in quality of life might be still significant [48]. Such awareness comes as a relief and as an affirmation that their health is getting under control. As a matter of fact, some of the refugees even mentioned feel proud to know that they were* “getting along with the disease.”* But some other participants felt helpless for not being able to cope with the drug therapy regimen at some point, as *“nothing was working, and I felt unable to go ahead. I just wanted to give up and forget about it” *(Aboriginal participant). Others were still in denial of their HIV diagnosis. In fact, denial has been discussed as a coping mechanism in terms of refusing to acknowledge an event or felling and forgetting that something ever happened [49]. Although coping with a stressful situation such as HIV diagnosis can promote change [50], some participants brought up issues of medication toxicity as a way to reinforce denial: *“I do not feel anything, really, it is like I have nothing. I do not want to take the meds as I know they are bad for you and might kill you anyway. I would not cope well with the side effects of them.” *(Latino participant). 

Resources were understood primarily in the context of availability and access, along with use and allocation. Individuals access those resources that are available to them, either as a refugee in a different country:

*“[t]hey offered us support at different levels, I remember. From “what to expect from Canada”, to adaptation to the new culture and where and how to learn English, all material and brochures in Spanish.” *

Or as a long-time, and historical, resident of Canada. Either way, the availability of resource information about HIV itself is not necessarily accessible or perceived as such due to improper allocation or delivery of information. For example, we heard from the Aboriginal groups that resources might be focused on specific services that are unlikely to be “one stop” shopping for every need. Financial support was a resource that was perceived as neither available nor properly allocated for housing, appropriate food, and clothing:

*“I was getting $40 for food allowance and $10 dollars for water supply, but they took it back because they told me “you are doing volunteer work at the gathering place, so, you are getting food and water already”… but you know that is not enough. I volunteer there once a week… food does not last that long.” (Aboriginal participant).*

*“I would like to see if those people [the government] could live with that amount for one month to pay for rent, food, transportation and clothing!” (Latino participant).*

When resources are scarce, some participants mentioned using local food banks and by-donation clothing stores, while others felt too proud, and sometimes ashamed, of using these services. Feelings of pride and shame might indeed prevent people from fully utilizing available resources, although this might be challenging to address, as this can be an ingrained personality trait. Another common reason for not utilizing existing resources is being unaware that they exist [51, 52], which might have been the case of some for some of our participants:

*“Really? I did not know that we had such services in Vancouver and that we could use them [when hearing that local organizations offer free haircuts, yoga and the Internet for HIV-positive individuals].” (Latino participant).*

Unawareness associated with confusion over health care and resources entitlements negatively influences the decision in utilizing such services, as also discussed by Thomas et al. [35] when studying immigrants in the UK Support, as well as the other two categories, was understood as influencing one another: one might experience empowerment upon feeling supported and one might utilize the resources available in a supportive manner [53]. Emotional, financial, and instrumental (e.g., resources) support were discussed conjointly as “*the individual belief that one is cared for and loved, esteemed and valued, and belongs to a network of communication and mutual obligations*” [54]. Similarly to resources, support was a perceived attribute that might entail caring for and appreciating the other, showing empathy, providing help, and so on. For some individuals, receiving support can be considered as an essential component towards engaging in disclosing one's HIV status, while others voiced that disclosing sexual orientation was not an issue compared to disclosing HIV status. The ability to comfortably disclose sexual orientation can be partially explained, at least for the Aboriginal participants, by the accepted notion of a two spirited person, for example, the belief in the existence of three genders: the male, the female, and the male-female gender. 

Religion and spirituality can play a rather important role as coping mechanisms for those facing illnesses. According to Siegel and Schrimshaw [55], people with HIV can become more spiritual in order to create an environment that allows them to ease the emotional burden of the disease. This practice also enhances and strengthens personal empowerment, self-control and sense of belonging. In addition, it has been suggested that spirituality helps HIV patients to accept their condition easier and to preserve their overall health for a longer period of time. Kudel et al. [56] also emphasized the fact that due to the constant social stigma around HIV, religion and spirituality can positively impact one's psychological health. Faith-based organizations and spiritual counsellors can be highly effective in improving the overall health of the HIV patients along with reducing the rate of HIV transmission and social stigma within their living environments. This is mainly due to the fact that people tend to perceive such organizations as places that offer a sense of security and social acceptance [57]. 

In our study, the impact of religion and spirituality surfaced while participants discussed the concept of emotional support. A participant from one of the Aboriginal groups got nods from others when he said: *“I pray to the Lord I'm alive every day… that I'm alive and moving on with my life.” *The Latinos discussed religion in more general terms when emphasizing the need to have faith and the belief that *“everything will be fine if you believe”*. Garcia and Parker [58] found that the interrelation of religious beliefs associated with individual networks can encourage HIV resources and services utilization and could be further explored within these groups.

We heard that most participants make effort to preserve an active social network around them and that the size of such network tends to remain relatively stable throughout life [59]. Although the groups did not discuss their number of friends or the size of their social networks per se, they did refer to the social support they get from various levels. For the Latinos in particular, a supportive network can be strongly associated with family ties, a commonality that binds them together even within the process of immigration and acculturation [60]. Although important, family ties were not prominent to our participants as per their refugee status in arriving “*with nothing, and families were left behind*” (see further discussion ahead). For them, as well as for others HIV seropositive individuals, the mobilization of a range of local supporting networks can have a constructive implication to their lives [61]. In this case, social capital surfaces related to networks as the sum of supportive relationships in a given community and a key player in developing individual coping and adaptive strategies in stressful environments. Social capital implies a system of interaction, resource sharing, and communication among individuals and groups, and it is also used in the context of solidarity [62]:

*“We are kind of alone here, on our own away from our families. We have to rely on each other, from the same culture, as we understand our traditions, the way we approach people, the values and beliefs we hold.” (Latino participant).*

Due to high levels of stigma, more frequently than not HIV patients became disconnected from their support networks of family members and partners. According to Knowlton and colleagues [61], when seropositive individuals obtain a certain level of emotional support from those who are also HIV positive, such network can create a rather fragile system when there is an imbalance between health and disease. We did not perceive or hear that this was necessarily the case for our participants, however. 

## 8. Concluding with Some Considerations

The scientific literature has plenty of information on the medical side of HIV, particularly the uptake of HIV antiretroviral therapy adherence, cost, availability, and side effects [3, 4]. However, the literature on access and use of nonmedical resources services is sparse. We have discussed some of the issues in accessing these nonmedical resources to support programs that integrate collaborative planning, education, and outreach, as well as internet-based interventions that would hold cultural sensitivity to marginalized populations [63, 64]. For example, the Aboriginal participants seemed to favour social relationships and the respect of friends, families, and community over their individual health. As a result, resource services should be tailored to fit this social and cultural context while acknowledging the consequences of lingering issues of stigma, racism, and poverty [65, 66].

## 9. “What Are the Educational and Service Needs of Those We Are Not Reaching?” 

This research question raised by a local HIV resource organization prompted our participants to identify ongoing needs in different areas, including:

- clean, safe, and affordable housing;
- clothing and culturally appropriate food;
- information on, and increased advocacy for, income assistance;
- culturally relevant and language-sensitive HIV education and awareness material;
- opportunities for exchange of information within culturally relevant and language-sensitive services and peer-led supporting groups and spaces;
- working opportunities;
- improved communication within and between patients, health professionals, and the community at large;
- faith-based organizations along with spiritual counselors.

It remains imperative that research within people living with HIV fosters egalitarian and participatory methods. Our collaborative research attempted to recognize the strengths and resilience of members from Aboriginal descent and refugees. The value of community-based research initiatives lies on its ability to give voice to, and collaboratively engage, community members that would have remained silent otherwise. As highlighted by Salmon et al. [11], the training given to the peer facilitators might have provided them with skills and experience for embracing future research, community involvement, and action. The findings from this small study can support the development of larger, more extensive follow-up community-based interventions that could also include females and other diverse gender-identified individuals.
